# Design Modifications of the Twin-Block Appliance: Facial and Dentoalveolar Outcomes in Class II Individuals

**DOI:** 10.3390/dj14100648

**Published:** 2026-10-08

**Authors:** Omar Maximino Milia, Mercedes Martín Moreno, Susana De la Cruz Vigo, Alberto-José López-Jiménez

**Affiliations:** 1 Advanced Orthodontics Master’s Program, European University of Madrid, C/Murallas de Lugo 23, 28300 Aranjuez, Spain; mercedeselvira.martin@universidadeuropea.es (M.M.M.); susana.delacruz@universidadeuropea.es (S.D.l.C.V.); 2 PhD Program, European University of Madrid, C/Murallas de Lugo 23, 28300 Aranjuez, Spain; 3 Orthodontic Teaching Unit, Francisco de Vitoria University, 28223 Madrid, Spain; 4 Dental Clinical Specialities Department, Complutense University of Madrid, 28040 Madrid, Spain; 5 Section of Orthodontics, Institution Mississippi of Madrid, Santísima Trinidad, 5, 28040 Madrid, Spain

**Keywords:** Class II malocclusion, Modified Twin Block, functional appliance, cephalometry, mandibular retrognathia, craniofacial growth, orthodontics

## Abstract

**Objective:** This study aimed to evaluate and compare the sagittal and vertical cephalometric changes observed in growing patients with Class II malocclusion and permanent dentition treated with a Modified Twin Block (TBM) with those observed in a historical control group of untreated individuals. **Materials and Methods:** An observational, comparative, longitudinal, retrospective study was conducted using 240 lateral cephalometric radiographs from 120 individuals with Class II malocclusion and mandibular retrusion relative to the maxilla. The treatment group (TG) comprised 60 patients previously treated with the Modified Twin Block (TBM), aged 11.5–14 years at the initial assessment (T1), whereas the control group (CG) comprised 60 untreated individuals retrospectively selected from historical records in the AAOF Craniofacial Growth Legacy Collection. Two cephalometric records were analysed for each individual (T1 and T2). Eleven cephalometric variables representing the sagittal, vertical, and angular dimensions included in Areas 1, 2, and 5 of the cephalometric protocol were evaluated. Changes were calculated as T2 − T1 and compared between groups for the overall sample and after stratification by sex. The *p* values for the 33 comparisons were adjusted using the Benjamini–Hochberg procedure to control the false discovery rate. Between-group mean differences, 95% confidence intervals, and effect sizes using Hedges’ g were also calculated. As a sensitivity analysis, linear regression models were fitted adjusting for the corresponding baseline cephalometric value, age at T1, sex, and the duration of the T1–T2 interval, with HC3 robust standard errors. **Results:** No statistically significant age differences were observed between groups at T1 or T2. However, the T1–T2 observation interval was significantly longer in the control group than in the treatment group (1.80 ± 0.31 vs. 1.65 ± 0.26 years; *p* = 0.004). After Benjamini–Hochberg correction of the 33 comparisons, statistically significant between-group differences remained for seven variables in the overall sample. In the multivariable sensitivity analysis, after adjustment for the baseline cephalometric value, age at T1, sex, and observation-interval duration, followed by correction for multiple comparisons, differences remained significant for A-N, S-Go, A-Me, N-Me, and ANB. Adjusted TG − CG differences were −1.70 for A-N (95% CI: −2.36 to −1.04), +2.29 for S-Go (95% CI: 1.04 to 3.53), +1.50 for A-Me (95% CI: 0.53 to 2.47), +3.49 for N-Me (95% CI: 2.01 to 4.96), and −1.04° for ANB (95% CI: −1.46 to −0.62). No significant differences were observed in functional occlusal plane inclination. Interaction analyses provided no statistically significant evidence of differences between males and females in treatment-group-associated changes. **Conclusions:** In growing patients with Class II malocclusion and permanent dentition, TBM treatment was associated with differences in sagittal and vertical cephalometric changes compared with the historical control group. The most consistent findings involved A-N, S-Go, A-Me, N-Me, and ANB and remained significant after multivariable adjustment and correction for multiple comparisons. These results should be interpreted in light of the retrospective, non-randomised design, the use of historical controls, the absence of a homogeneous indicator of skeletal maturation, and the lack of long-term follow-up.

## 1. Introduction

Class II malocclusion is a common dentoskeletal disorder during growth and may be associated with different combinations of sagittal maxillomandibular discrepancies. Among these, mandibular retrusion relative to the maxilla is a common clinical presentation and may be accompanied by alterations in the occlusal relationship and facial profile. Treatment during growth aims to correct or reduce these discrepancies by taking advantage of craniofacial growth potential and promoting a more favourable maxillomandibular relationship [1,2,3,4,5].

Functional appliances have been widely used to treat growing patients with Class II malocclusion associated with mandibular retrusion. Among them, the Twin Block, originally developed by William J. Clark [3,6,7,8,9,10], is one of the most extensively studied removable functional appliances. Its mechanism is based on inclined planes that modify the functional mandibular position during appliance wear. Several studies have evaluated its dentoalveolar and skeletal effects using lateral cephalometric radiographs and, more recently, three-dimensional techniques.

The work of Jeffrey Okeson [11] has been taken into account, particularly with regard to all aspects related to the perioral musculature and the temporomandibular joints, and specifically to constructive bite registration.

Available evidence indicates that treatment with Twin-Block-type functional appliances may be associated with changes in the sagittal maxillomandibular relationship, including reductions in the ANB angle, changes in sagittal maxillary displacement, and changes in various mandibular and vertical dimensions. However, the magnitude and nature of these changes vary among studies, partly because of differences in sample characteristics, growth and maturation stage, treatment duration, appliance design, and cephalometric methods.

A modified version, termed the Modified Twin Block (TBM), was developed from the original Twin-Block design, incorporating modifications intended to facilitate specific clinical objectives and reduce certain undesirable dentoalveolar effects. These modifications include features aimed at controlling lower-incisor inclination, managing the posterior vertical dimension, and, when indicated, integrating maxillary expansion procedures. The appliance can be used with different manufacturing approaches, including conventional and digital designs. However, the present study evaluates changes associated with TBM treatment as a whole and was not designed to determine whether these modifications provide additional advantages over the conventional Twin Block or to directly compare the different TBM modalities [12,13].

Cephalometric evaluation of changes occurring during growth requires, as far as possible, differentiation between treatment-associated changes and those resulting from spontaneous craniofacial growth. For this reason, comparison with untreated individuals is important for interpreting changes observed during the treatment and observation period. In addition, reproducibility of landmarks and cephalometric measurements is essential when the differences under analysis are small.

In this context, the aim of the present study was to evaluate and compare sagittal, vertical, and angular cephalometric changes observed in growing patients with Class II malocclusion and permanent dentition treated with the Modified Twin Block (TBM) with those observed in a historical control group of untreated individuals. Eleven variables corresponding to Areas 1, 2, and 5 of a broader cephalometric protocol were analysed.

The null hypothesis (H0) stated that there would be no statistically significant differences between the treatment and control groups in T2 − T1 changes in the cephalometric variables evaluated. The alternative hypothesis (H1) stated that there would be statistically significant differences between the two groups in at least one of these variables.

## 2. Materials and Methods

### 2.1. Study Design

The present study was designed as an observational, comparative, longitudinal, and retrospective study.

The sample comprised 240 lateral cephalometric radiographs obtained from 120 individuals of both sexes with mandibular retrusion relative to the maxilla. All included individuals were growing and had permanent dentition in accordance with the established eligibility criteria.

A minimum age of 11.5 years was established for both groups. The treatment group (TG) included 60 patients treated with the TBM appliance, with a maximum age of 14 years at treatment onset (T1). Because the treatment and observation period was approximately 1.5 years, the final assessment (T2) could occur up to approximately 15.5 years of age.

The control group (CG) comprised 60 untreated individuals for whom two lateral cephalometric radiographs corresponding to T1 and T2 were available. CG data were obtained from a historical database; consequently, the intervals between the two radiographs had not been prospectively established with a uniform duration but depended on the timing of the original radiographic assessments. For the present study, pairs of radiographs corresponding to the defined observation period were selected, with a maximum age of 15.5 years at T2.

Thus, the age limits of 14 and 15.5 years refer to different points in the observation period: 14 years represents the maximum age at treatment onset in the TG (T1), whereas 15.5 years represents the maximum age at the final assessment in the CG (T2). Age comparability between groups at T1 and T2, as well as the duration of the T1–T2 observation interval, was subsequently evaluated statistically.

#### 2.1.1. Eligibility Criteria

The inclusion criteria established for the treatment group (TG) and control group (CG) were as follows:

Individuals of both sexes.
Minimum age of 11.5 years at treatment onset or initial assessment (T1).
Class II molar relationship, defined as a discrepancy greater than 3 mm between the most posterior limit of the maxillary molar and the most posterior limit of the mandibular molar; smaller discrepancies were considered Class I molar relationships. This determination was made using the cephalometric method developed specifically for this study, based exclusively on the molar relationships observed on the radiographs and without considering clinical data.
ANB angle greater than 0° (angle formed by points A, N, and B).
Complete or nearly complete permanent dentition through the first permanent molars, with or without eruption of the second permanent molars.
Presence of premolars in both arches and mandibular canines in the oral cavity, even if root apices were not fully developed.
Presence of maxillary canines in the oral cavity, except in individuals with impacted canines for whom treatment had been initiated, provided that this did not compromise the stability of the maxillary component of the appliance.
Presence of anterior overbite (absence of anterior open bite).

The exclusion criteria were as follows:

Age below 11.5 years for both groups.
Age greater than 14 years at the initial assessment (T1) in the treatment group or greater than 15.5 years at the final assessment (T2) in the control group.
ANB angle below 0°.
Presence of anterior open bite.
Class I molar relationship.
Class III molar relationship.
Lateral cephalometric radiographs obtained without occlusal contact.
Permanent dentition not complete through the first molars in both arches.

#### 2.1.2. Ethical Considerations

The study was conducted in accordance with the Declaration of Helsinki and was approved by the Ethics Committee of Universidad Europea de Madrid (CI 2025-130) on 27 May 2025. Written informed consent was obtained from all participants for the use of their radiographic images for diagnostic purposes.

### 2.2. Modified Twin-Block (TBM) Methodology

The original appliance design was modified with the aim of improving treatment outcomes and facilitating clinical management, but not with the intention of establishing differences relative to the original design; this issue will be addressed in a future study.

**Figure 1:**
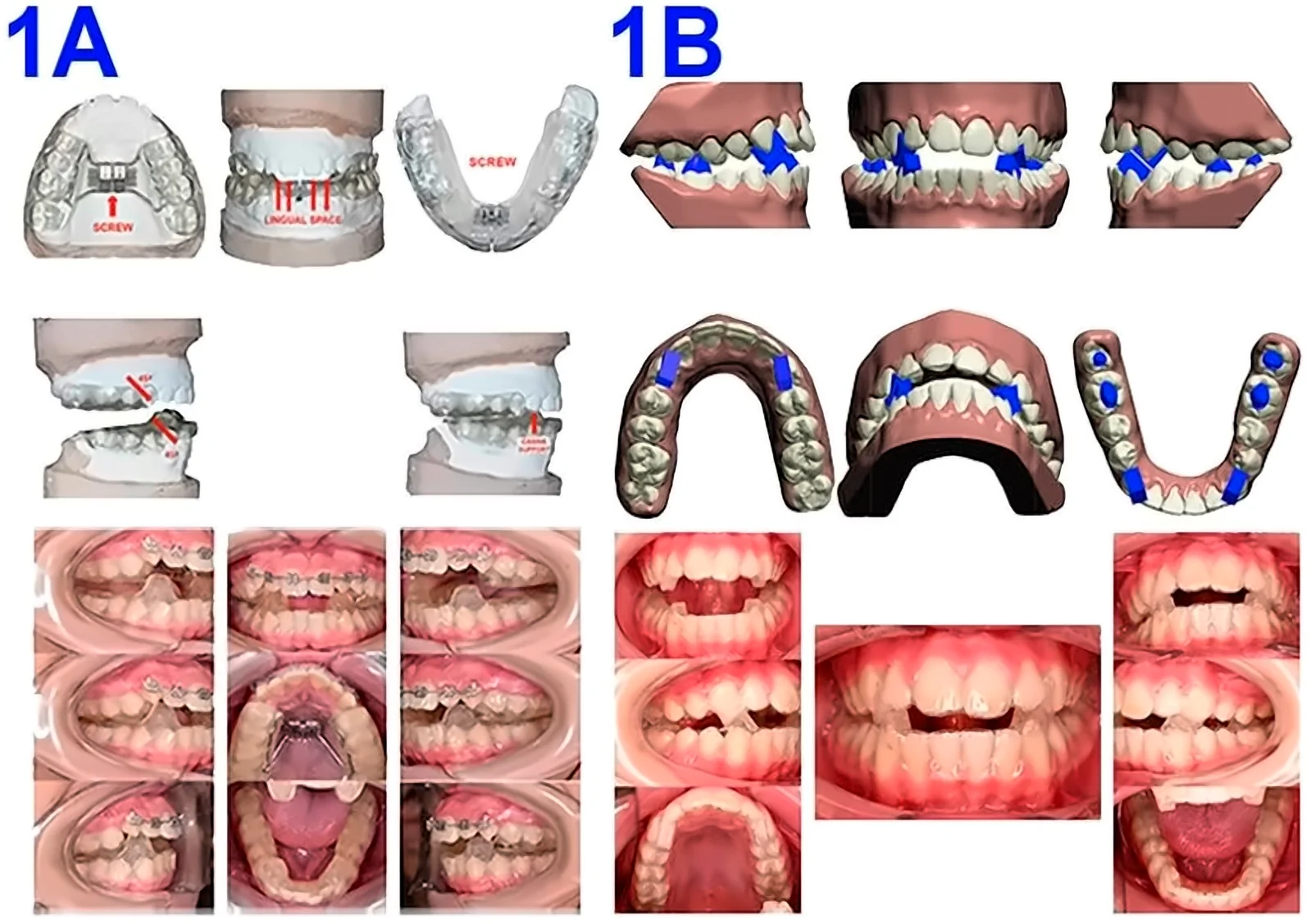
(**A**) Modified Twin-Block; (**B**) wings of aligners.

The maxillary component of the original appliance was modified in two ways. First, when rapid maxillary expansion was indicated, clear acrylic splints were placed bilaterally to allow cementation and were connected by a Hyrax screw (Figure 1A). Second, when expansion was performed with clear aligners, a specific design developed for this study was used, incorporating inclined planes into the aligners (Figure 1B).

The mandibular component covered the entire arch and was designed in two different forms. When splints were used in the maxillary arch, the mandibular appliance was fabricated from the same material, covering the entire mandibular arch and incorporating a transverse screw to permit dentoalveolar expansion. Unlike the original design proposed by the author, full coverage of the mandibular arch was intended to prevent labial proclination of the lower incisors. Anterior to the lower inclined planes, support areas for the maxillary canines were created, with inclined surfaces on each side to facilitate canine guidance and sliding (Figure 1A).

Conversely, when clear aligners were used in the maxillary arch, corresponding elements with matching inclined planes were incorporated into the mandibular aligners. 3Shape Ortho Analyzer software (version 2021.1, Copenhagen, Denmark) was used for the digital design of all patients treated with clear aligners, and the appliances were digitally fabricated (Figure 1B).

Several aspects of clinical management should also be highlighted because of the intended therapeutic effects. One was the prescribed appliance wear time, set at a minimum of 12 months and a maximum of 18 months, for 22 h per day. Based on clinical experience, these conditions were considered appropriate to optimise the therapeutic effects while minimising relapse.

Another important aspect was the clinical protocol used to correct the posterior open bite that may develop during treatment with the appliance.

This protocol was intended to permit extrusion of the maxillary posterior teeth, with the aim of producing anterior mandibular rotation while simultaneously reducing functional occlusal plane inclination.

A relevant element of the procedure was the construction-bite registration, for which the following criteria were established:

- Maximum sagittal advancement of 4 mm at canine level, with the dental midlines centred, provided that correction of the transverse discrepancy did not exceed 2 mm. If greater advancement or additional midline correction was required, adjustments were made at 4-month intervals without exceeding the limits described above.
- Vertical bite opening was set at 3–4 mm in the posterior region, at the point where the two arches were closest to each other, thereby providing sufficient thickness for both components of the appliance.

### 2.3. Cephalometric Method

This represented a major challenge at the beginning of the study because the radiographic records obtained from patients in the treatment group (TG) were acquired under ideal conditions, using the same operator and the same radiographic equipment. In contrast, the radiographs corresponding to individuals in the control group (CG), obtained from the “AAOF Craniofacial Growth Legacy Collection” of the American Association of Orthodontists Foundation, only provided a calibration reference and confirmation that all scans had been digitized at the same scale (100%).

All radiographs were rigorously calibrated and standardised prior to performing the measurements. For this purpose, the calibration scales incorporated into the images at the time of acquisition were used. Radiographs lacking these calibration elements were excluded, as were those in which the calibration scale could not be clearly visualised without distortion.

The situation described above made it necessary to establish a positional reference that could be applied consistently to both groups of individuals.

### 2.4. S–N Positional Reference Plane

- Nasion (N): Anterior point of the frontonasal suture.
- Sella (S): Geometric center of the sella turcica.

**Figure 2:**
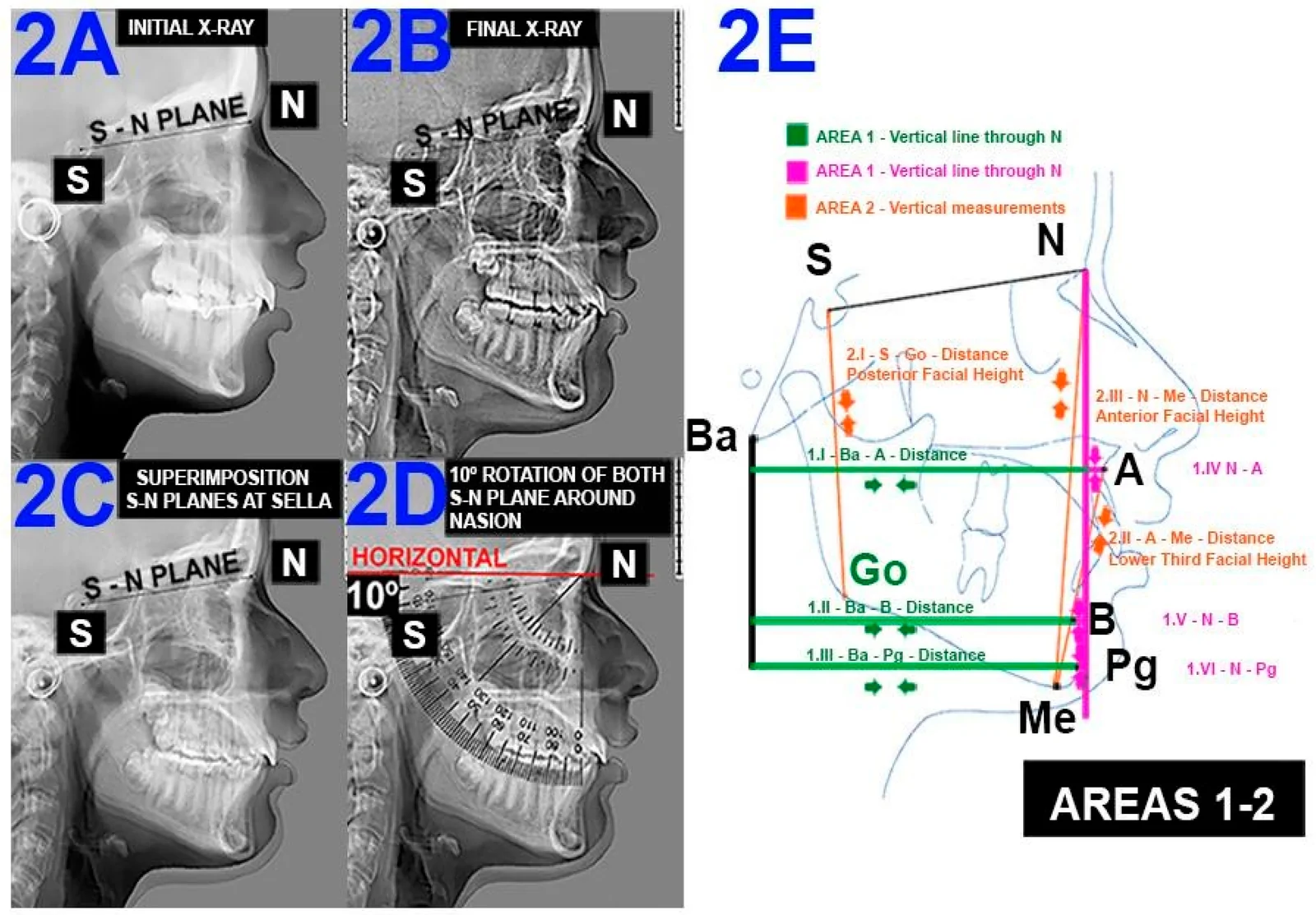
(**A**) Initial telerradiographic; (**B**) Final telerradiographic; (**C**) Superimposition of both S–N planes; (**D**) Rotation of S-N plane around nasion; (**E**) Cephalometric Measurements.

The same procedure was performed for each patient whose initial and final radiographs were to be compared. Figure 2A (initial) and Figure 2B (final) correspond to the same treated patient and are presented as an example. In both radiographs, the previously described plane was traced. Superimposition of both S–N planes at point S was required (Figure 2C). It is highly probable that the N point on the final lateral cephalometric radiograph might not coincide with that of the initial radiograph due to craniofacial growth.

After superimposition of the lateral radiographs, both images were simultaneously repositioned until the superimposed reference planes formed an inclination of 10° relative to a horizontal plane passing through the N point in both radiographs or, when growth had occurred, through the N point of the final radiograph (Figure 2D).

Although the measurements were performed separately for each individual, this final procedure provided homogeneity to the measurement method. Subsequently, the superimposed, calibrated, and angulated radiographs (initial and final) of each patient were individually imported into a cephalometric software program to perform the corresponding measurements.

### 2.5. Cephalometric Measurements

**Area 1: Horizontal Linear Measurements of Hard Tissues (Figure 2E)**

**(I) Maxillary length (Ba–A)**

Distance between a vertical line descending from the basion point and a perpendicular line passing through point A.

**(II) Lower alveolar length (Ba–B)**

Distance between a vertical line descending from the basion point and a perpendicular line passing through point B.

**(III) Mandibular length (Ba–Pg)**

Distance between a vertical line descending from the basion point and a perpendicular line passing through point Pg.

**(IV) Sagittal position of point A**

Distance between point A and the vertical line descending from point N, measured on a horizontal line passing through point A.

**(V) Sagittal position of point B relative to the N vertical**

Distance between point B and the vertical line descending from point N, measured on a horizontal line passing through point B.

**(VI) Sagittal position of point Pg relative to the N vertical**

Distance between pogonion (Pg) and the vertical line descending from point N, measured on a horizontal line passing through point Pg.

**Area 2: Vertical Linear Measurements of Hard Tissues (Figure 2E)**

**(I) Posterior facial height (S–Go)**

Distance between sella (S) and gonion (Go).

**(II) Anterior maxillary height (A–Me)**

Distance between point A and menton (Me).

**(III) Anterior facial height (N–Me)**

Distance between nasion (N) and menton (Me).

**Figure 3:**
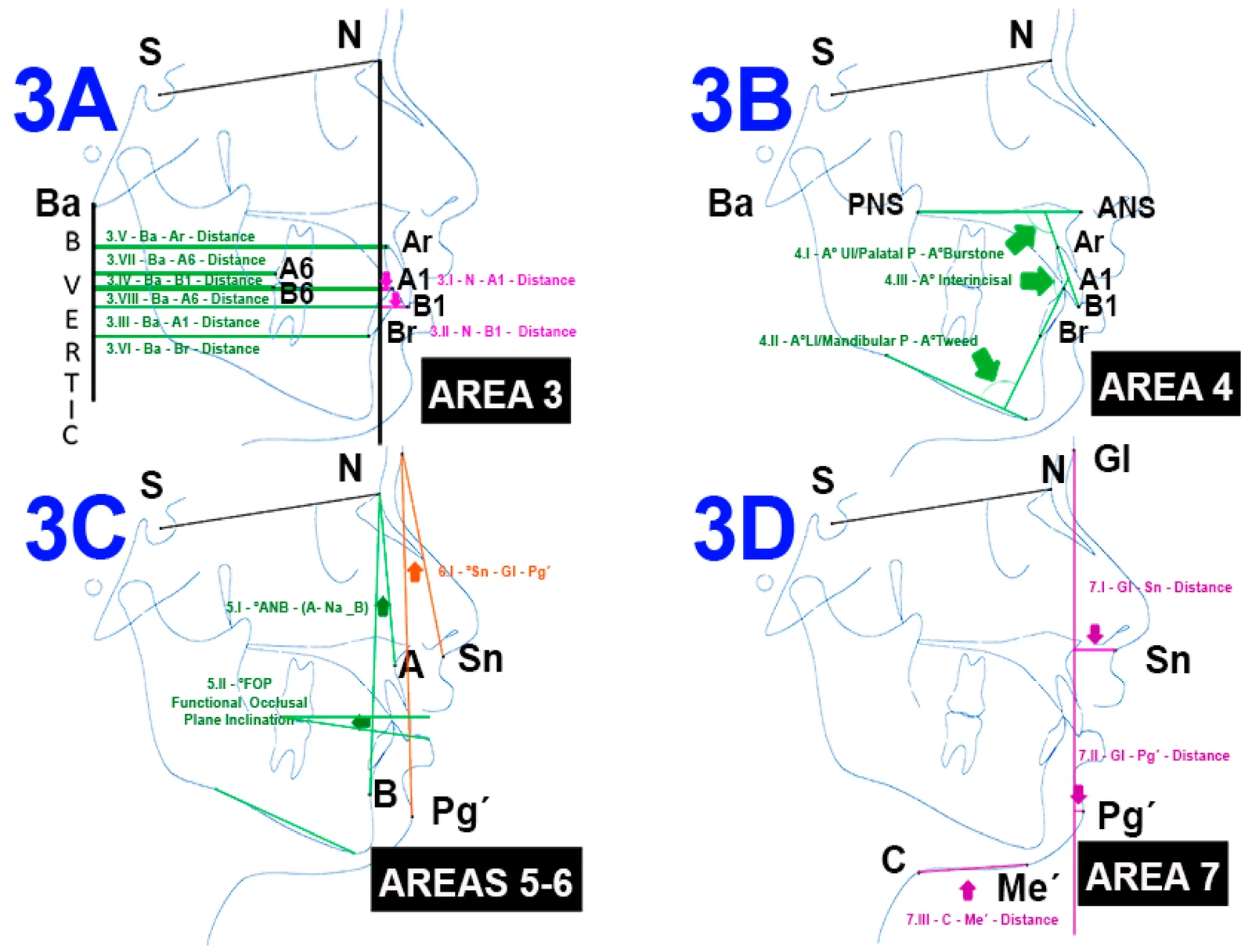
Cephalometric Measurements. (**A**) Area 3; (**B**) Area 4; (**C**) Areas 5–6; (**D**) Area 7.

**Area 3: Horizontal Dental Measurements (Figure 3A)**

**(I) Distance from N vertical to point A1**

Distance between the vertical line descending from point N and the incisal edge of the upper incisor (point A1).

**(II) Distance from N vertical to point B1**

Distance between the vertical line descending from point N and the incisal edge of the lower incisor (point B1).

**(III) Distance Ba–A1**

Distance between a vertical line descending from the basion point and a perpendicular line passing through point A1 (upper incisor edge).

**(IV) Distance Ba–B1**

Distance between a vertical line descending from the basion point and a perpendicular line passing through point B1 (lower incisor edge).

**(V) Distance Ba–Ar**

Distance between a vertical line descending from the basion point and a perpendicular line passing through point Ar (upper incisor apex).

**(VI) Distance Ba–Br**

Distance between a vertical line descending from the basion point and a perpendicular line passing through point Br (lower incisor apex).

**(VII) Distance Ba–A6**

Distance between a vertical line descending from the basion point and a perpendicular line passing through point A6 (most distal point of the upper first molar).

**(VIII) Distance Ba–B6**

Distance between a vertical line descending from the basion point and a perpendicular line passing through point B6 (most distal point of the lower first molar).

**Area 4: Angular Dental Measurements (Figure 3B)**

**(I) Palatal plane–upper incisor axis angle**

Angle formed by the palatal plane (PNS–ANS) and the long axis of the upper incisor (A1–Ar).

**(II) Mandibular plane–lower incisor axis angle**

Angle formed by the mandibular plane (tangent to the mandibular basal border passing through point Me) and the long axis of the lower incisor (B1–Br).

**(III) Interincisal angle**

Angle formed by the long axes of the upper and lower incisors.

**Area 5: Angular Measurements of Hard Tissues (Figure 3C)**

**(I) ANB angle**

Angle formed by the N–A and N–B planes.

**(II) Inclination of the functional occlusal plane**

Angle formed between the functional occlusal plane and a horizontal reference plane.

**Area 6: Angular Measurements of Soft Tissues (Figure 3C)**

**(I) Sn–Gl–Pg’**

Angle formed by the Gl–Pg’ and Gl–Sn planes.

**Area 7: Horizontal Linear Measurements of Soft Tissues (Figure 3D)**

**(I) Gl vertical to Sn**

Distance from the vertical line descending from glabella (Gl) to subnasale (Sn).

**(II) Gl vertical to Pg’**

Distance from the vertical line descending from glabella (Gl) to soft tissue pogonion (Pg’).

**(III) Distance C–Me’**

Distance between the cervical point (C) and soft tissue menton (Me’).

A cephalometric protocol comprising 26 horizontal, vertical, and angular measurements of hard tissues, soft tissues, and dental structures was developed for the overall study. For the purposes of the present manuscript, only 11 of these parameters were selected for analysis: Ba-A, Ba-B, Ba-Pg, A-N, B-N, Pg-N, S-Go, A-Me, N-Me, ANB, and Functional Occlusal Plane Inclination.

## 3. Statistical Analysis

Data were collected and organised in a Microsoft Excel database. Statistical analyses were performed according to the procedures described below.

### 3.1. Assessment of Reproducibility (Measurement Error)

The measurements obtained were compared with those made by another operator and were organised into three groups: “Operator 1” (lead author of the article), “Operator 2” (second author of the article), and “Operator 1st–2nd measurement” (second measurement performed by the lead author).

The comparison was performed for three pairs: “Operator 1/Operator 2”, “Operator 2/Operator 1st–2nd measurement”, and “Operator 1/Operator 1st–2nd measurement”.

Reproducibility was assessed using Lin’s concordance correlation coefficient. This coefficient evaluates agreement between two sets of measurements with reference to the line of identity. The coefficient was calculated pairwise for each variable, combining treated and untreated cases.

The values obtained showed high concordance. For all variables analysed, the concordance coefficient exceeded 0.90, close to the maximum possible value of 1.

### 3.2. Descriptive and Comparative Analysis

The distribution of quantitative variables was assessed using the Shapiro–Wilk and Kolmogorov–Smirnov tests. Comparisons between the treatment and control groups were performed using Student’s *t*-test when a parametric procedure was appropriate and the Mann–Whitney U test for variables analysed using non-parametric procedures.

Cephalometric changes were calculated as the difference between measurements obtained at T2 and T1 (T2 − T1). Comparisons of these changes between the treatment and control groups were performed for the overall sample and, additionally, after stratification by sex.

To control the increased risk of false-positive findings arising from multiple comparisons, the *p* values corresponding to the 33 comparisons—11 cephalometric variables in the overall sample, 11 in males, and 11 in females—were adjusted using the Benjamini–Hochberg procedure to control the false discovery rate (FDR).

In addition to *p* values, mean differences between the treatment and control groups, their 95% confidence intervals (95% CIs), and standardised effect sizes using Hedges’ g were calculated.

Age comparability between the treatment and control groups was assessed at T1 and T2 for both the overall sample and the sex-stratified analyses. The duration of the T1–T2 observation interval was also compared between groups.

Given the non-randomised nature of the study and the use of a historical control group, additional multivariable sensitivity analyses were performed to assess the robustness of the findings. For each cephalometric variable, the T2 − T1 change was modelled using linear regression, with study group as the main explanatory variable and adjustment for the corresponding baseline (T1) cephalometric value, age at T1, sex, and duration of the T1–T2 observation interval. In sex-stratified analyses, sex was not included as a covariate. HC3 robust standard errors were used to estimate confidence intervals and *p* values. The *p* values for the group effect obtained from these models were also adjusted using the Benjamini–Hochberg procedure, considering the 33 comparisons jointly. 

Potential effect modification by sex was additionally assessed using interaction terms between study group and sex. These analyses were used to determine whether there was statistical evidence of differences between males and females in the effect associated with treatment.

Statistical significance was set at *p* < 0.05. In analyses subjected to multiple-comparison correction, statistical significance was determined using the Benjamini–Hochberg-adjusted *p* value (<0.05).

## 4. Results

**Figure 4:**
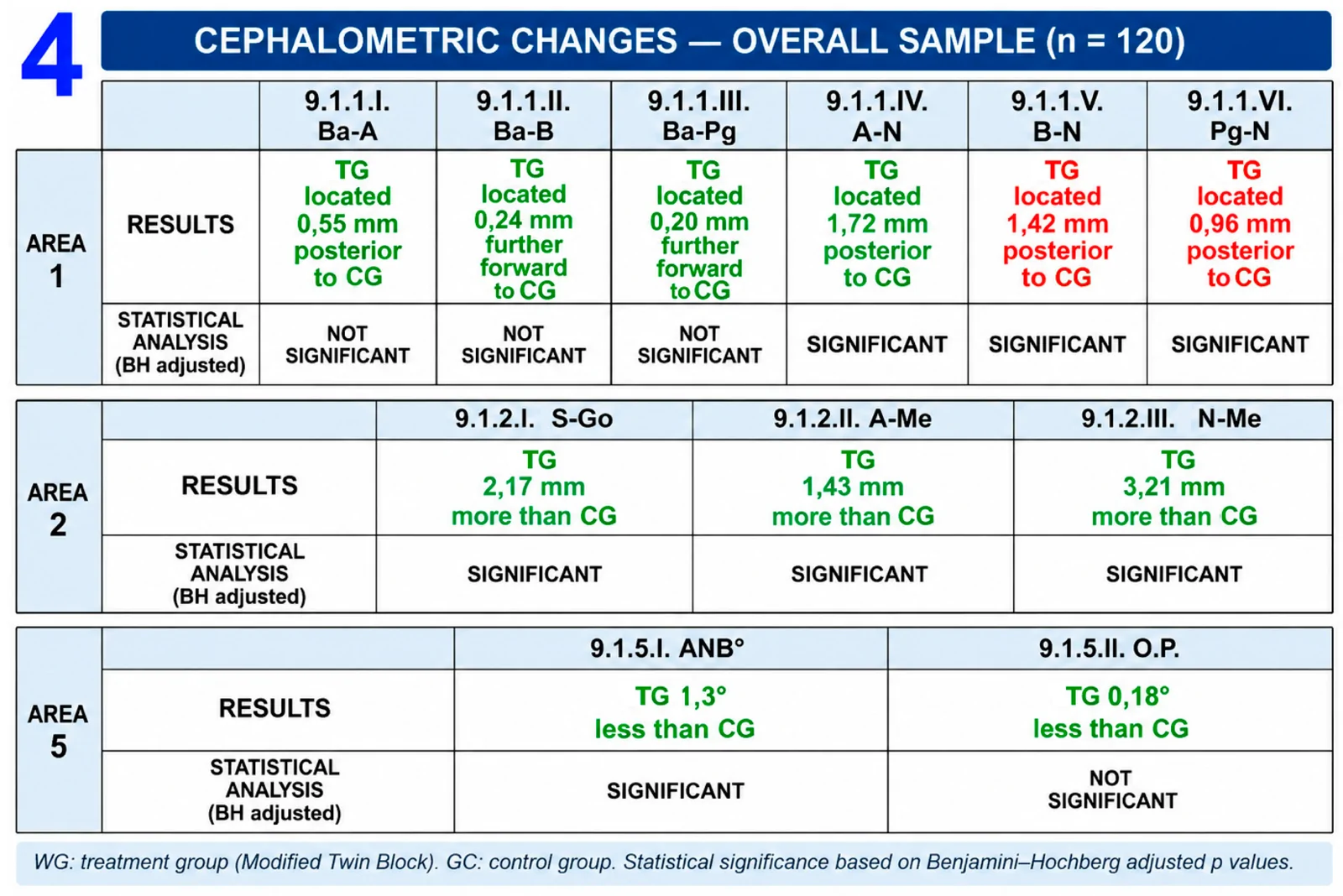
Cephalometric changes.

**Figure 5:**
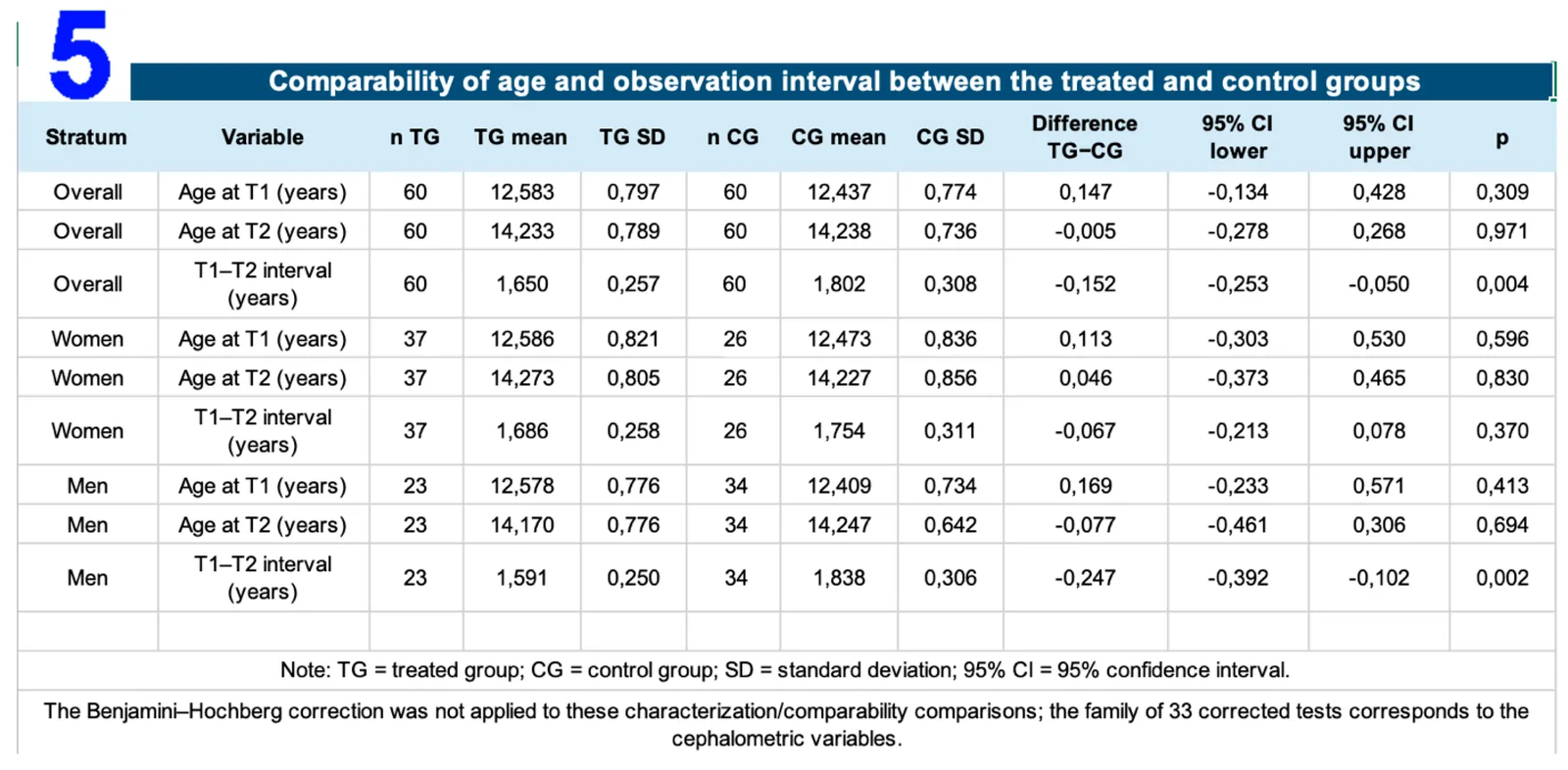
Comparability of age between the treated and control groups.

The results for the overall sample are presented in Figure 4 and Figure 5.

In Area 1, changes favourable to the correction of the Class II sagittal relationship are shown in green (positive +), whether they reflect less pronounced sagittal maxillary growth or greater sagittal mandibular development. Changes in the opposite direction are shown in red (negative −).

In Area 2, values shown in green (positive +) indicate a greater increase in the vertical dimensions assessed in the treatment group compared with the control group, whereas values shown in red (negative −) indicate changes in the opposite direction.

In Area 5, changes in the ANB angle and functional occlusal plane (OP) were assessed. A reduction in the ANB angle represents a decrease in the maxillomandibular sagittal discrepancy associated with Class II malocclusion. Regarding the inclination of the functional occlusal plane, a decrease in the angle would be consistent with anterior mandibular rotation and, therefore, with a favourable change in the sagittal relationship.

### 4.1. Group Comparability

Mean age at T1 was 12.58 ± 0.80 years in the treatment group (TG) and 12.44 ± 0.77 years in the control group (CG), with no statistically significant difference between groups (*p* = 0.309; mean difference = 0.15 years; 95% CI: −0.14 to 0.43). At T2, mean ages were 14.23 ± 0.79 years in the TG and 14.24 ± 0.74 years in the CG (*p* = 0.971). No statistically significant age differences between groups were observed at T1 or T2 in the sex-stratified analyses.

The mean duration of the T1–T2 observation interval was 1.65 ± 0.26 years in the TG and 1.80 ± 0.31 years in the CG, and this difference was statistically significant (*p* = 0.004). In the sex-stratified analysis, the difference in interval duration was not statistically significant among females (1.69 ± 0.26 vs. 1.75 ± 0.31 years; *p* = 0.370), whereas it was significant among males (1.59 ± 0.25 vs. 1.84 ± 0.31 years; *p* = 0.002).

### 4.2. Comparison of Cephalometric Changes

The analysis of T2 − T1 changes comprised 33 comparisons corresponding to the 11 cephalometric variables evaluated in the overall sample and in the male and female subgroups. Seventeen comparisons initially yielded nominal *p* values < 0.05. After application of the Benjamini–Hochberg procedure for multiple-comparison correction, 16 remained statistically significant. The only initially significant comparison that lost significance after correction was A-Me in the male subgroup.

**Figure 6:**
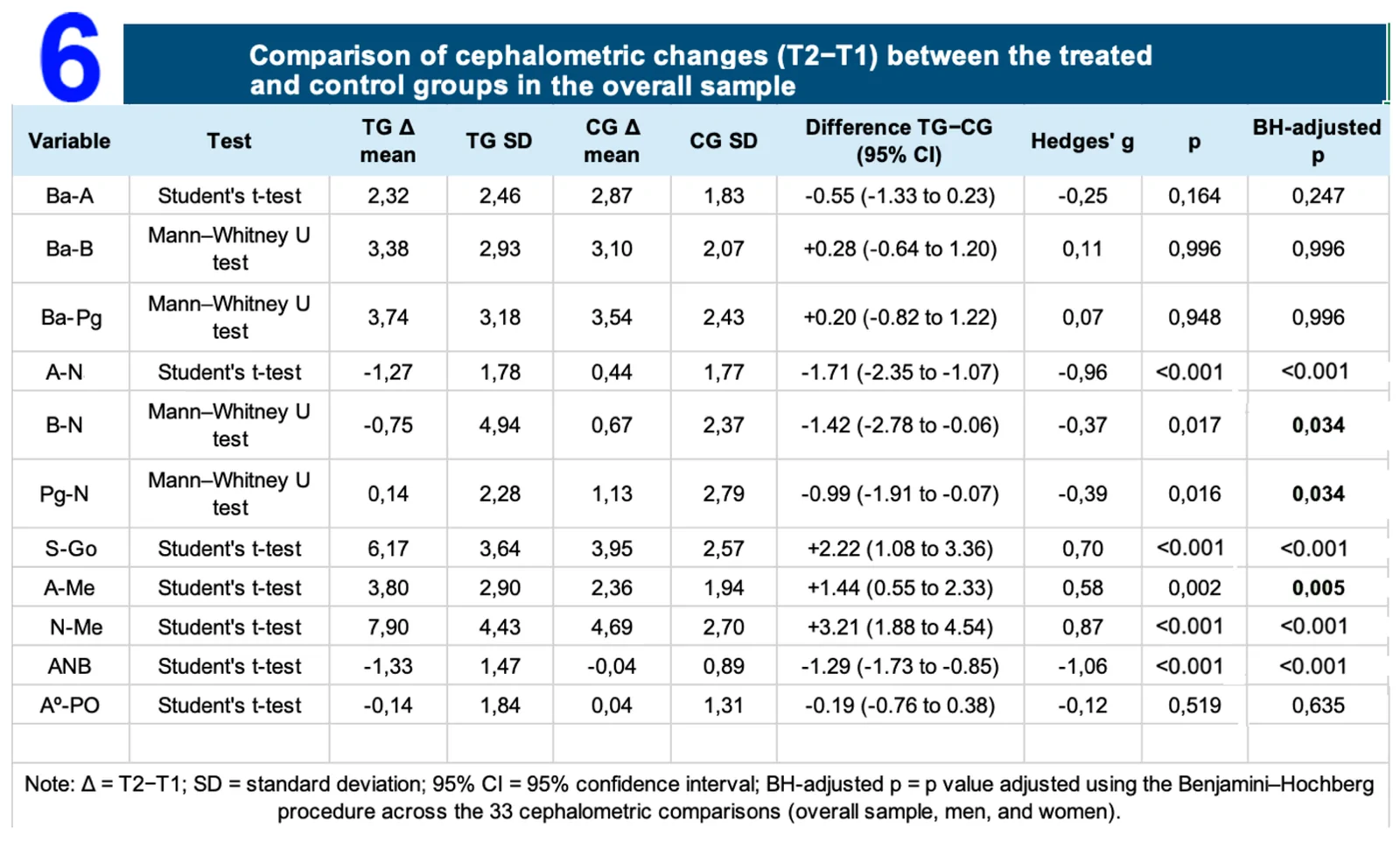
Comparison of cephalometric changes (T2-T1).

In the overall sample, differences between the TG and CG remained statistically significant after Benjamini–Hochberg correction for A-N, B-N, Pg-N, S-Go, A-Me, N-Me, and ANB (Figure 6).

Among the main findings in the overall sample, the mean TG − CG difference was −1.71 for A-N (95% CI: −2.35 to −1.07; Hedges’ g = −0.96), +2.22 for S-Go (95% CI: 1.08 to 3.36; g = 0.70), +1.44 for A-Me (95% CI: 0.55 to 2.33; g = 0.58), +3.21 for N-Me (95% CI: 1.88 to 4.54; g = 0.87), and −1.29° for ANB (95% CI: −1.73 to −0.85; g = −1.06). These findings show that statistically significant differences in several of the principal variables were accompanied by moderate-to-large effect sizes.

### 4.3. Multivariable Sensitivity Analysis

Given the retrospective, non-randomised nature of the study and the use of a historical control group, a multivariable sensitivity analysis was performed to assess the robustness of the findings to potential between-group differences.

After adjustment for the corresponding baseline cephalometric value at T1, age at T1, sex, and the duration of the T1–T2 observation interval, and after Benjamini–Hochberg correction for multiple comparisons, statistically significant differences in the overall sample remained for A-N, S-Go, A-Me, N-Me, and ANB.

The adjusted effects associated with the treatment group were −1.70 for A-N (95% CI: −2.36 to −1.04), +2.29 for S-Go (95% CI: 1.04 to 3.53), +1.50 for A-Me (95% CI: 0.53 to 2.47), +3.49 for N-Me (95% CI: 2.01 to 4.96), and −1.04° for ANB (95% CI: −1.46 to −0.62).

By contrast, the differences observed for B-N and Pg-N in the unadjusted analysis did not remain statistically significant after multivariable adjustment and correction for multiple comparisons.

### 4.4. Sex-Stratified Analysis

**Figure 7:**
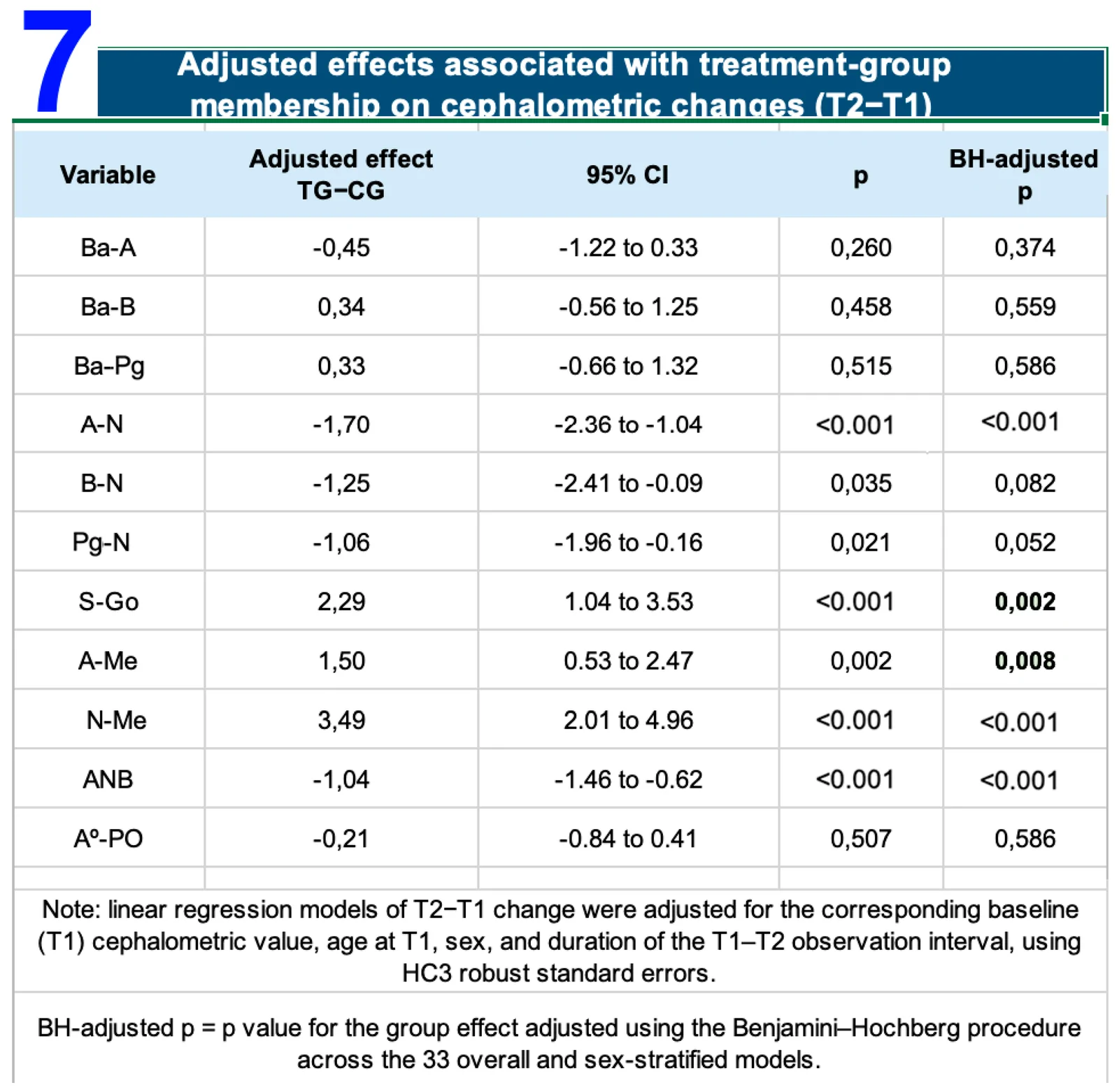
Adjusted effects associated with treatment-group membership on cephalometric changes.

In the sex-stratified multivariable analyses, A-N and ANB remained statistically significant after Benjamini–Hochberg correction in the male subgroup. In the female subgroup, A-N, S-Go, A-Me, N-Me, and ANB remained statistically significant after multivariable adjustment and correction for multiple comparisons. The results of the multivariable sensitivity analyses are presented in Figure 7.

However, formal group-by-sex interaction analyses provided no statistically significant evidence that treatment-group-associated changes differed between males and females. Accordingly, sex-stratified findings should be interpreted as exploratory analyses rather than as evidence of a sex-specific differential treatment response.

### 4.5. Interpretation of Results by Cephalometric Area

In Area 1, the most consistent finding was observed for A-N, with a smaller maxillary sagittal change in the TG than in the CG. This difference remained after both multiple-comparison correction and adjusted multivariable analysis. This finding is compatible with the so-called “headgear effect”, characterised by reduced sagittal maxillary displacement. Differences initially observed for other sagittal variables, such as B-N and Pg-N, were less robust after multivariable adjustment.

In Area 2, S-Go, A-Me, and N-Me showed greater increases in the TG than in the CG. These differences remained statistically significant after multiple-comparison correction and adjusted multivariable analysis, supporting the presence of between-group differences in changes in the vertical dimensions evaluated.

In Area 5, the change in the ANB angle showed a significantly greater reduction in the TG than in the CG. This finding remained after adjustment for baseline value, age, sex, and observation-period duration, as well as after correction for multiple comparisons, and was compatible with a reduction in the sagittal maxillomandibular discrepancy associated with Class II malocclusion.

By contrast, no statistically significant between-group differences were observed in the change in functional occlusal plane inclination (A–PO). Therefore, the data provided no evidence of a relevant differential treatment effect on this variable.

Figure 5 presents the comparability of age and observation-interval duration between the treatment and control groups; Figure 6 presents the comparison of T2 − T1 cephalometric changes between groups in the overall sample; and Figure 7 presents the adjusted comparison of T2 − T1 cephalometric changes between the treatment and control groups.

## 5. Discussion

### 5.1. Main Findings in Context

The main findings of this study show differences between the treatment and control groups in cephalometric changes involving sagittal and vertical dimensions. After multiple-comparison correction using the Benjamini–Hochberg procedure and, subsequently, in the multivariable sensitivity analysis adjusted for the baseline cephalometric value, age at T1, sex, and duration of the T1–T2 observation interval, the most consistent findings in the overall sample were observed for A-N, S-Go, A-Me, N-Me, and ANB.

In the sagittal dimension, the smaller A-N change observed in the treatment group than in the control group is compatible with the so-called “headgear effect”, characterised by reduced sagittal maxillary displacement. Likewise, the greater reduction in the ANB angle observed in the treatment group indicates a decrease in sagittal maxillomandibular discrepancy during the treatment and observation period. By contrast, although B-N and Pg-N showed statistically significant differences in the initial analysis of T2 − T1 changes, these differences did not remain significant after multivariable adjustment and correction for multiple comparisons and should therefore be interpreted with greater caution.

For the vertical component, the greater increases in S-Go, A-Me, and N-Me observed in the treatment group remained after the sensitivity analyses, indicating that the between-group differences in these dimensions were robust to adjustment for the covariates considered.

Overall, the direction of the observed pattern of changes is consistent with effects reported for the Twin Block and other functional appliances in recent systematic reviews and meta-analyses. These studies have reported reductions in the ANB angle, reduced sagittal maxillary displacement, and increases in different mandibular dimensions and facial height. Nevertheless, direct comparison of the magnitude of changes should be undertaken cautiously because studies differ in appliance design, sample characteristics, maturation stage, cephalometric variables, and treatment and observation duration.

### 5.2. Absence of a Differential Effect on the Functional Occlusal Plane

Although the clinical protocol was designed to promote posterior maxillary dentoalveolar extrusion and anterior mandibular rotation, no statistically significant between-group differences were observed in the change in functional occlusal plane (OP) inclination. This finding warrants specific consideration because one of the proposed clinical mechanisms of the appliance relates precisely to posterior vertical control and its potential influence on mandibular rotation.

Among the possible explanations, posterior dentoalveolar changes that may have occurred during treatment could have contributed to clinical closure of the posterior open bite without producing a change in functional occlusal plane inclination of sufficient magnitude to be detected cephalometrically during the period evaluated. In addition, growth-related eruptive changes in the control group may have reduced the observed between-group difference. Finally, the appliance wear period, approximately 12–18 months, may not have been sufficient for vertical dentoalveolar changes to translate into a detectable change in functional occlusal plane inclination.

### 5.3. A Digital Design Alternative in the Context of the Still-Limited Evidence on Functional Therapy with Aligners

The aligner-integrated inclined-plane design evaluated in this study, developed to permit both maxillary expansion and a fully digital workflow, should be considered in the context of the recent, although still limited, literature comparing aligner systems with mandibular-advancement mechanisms against the conventional Twin Block.

Available comparative studies suggest that aligner systems incorporating integrated occlusal ramps may exhibit different biomechanical behaviour from mandibular advancement systems based on buccal wings (*precision wings*), particularly with regard to posterior vertical control. These differences may be related to the specific design characteristics of each system.

From this perspective, the posterior inclined-plane mechanism incorporated into our design is biomechanically more similar to functional aligner appliances using a comparable posterior architecture than to systems based exclusively on buccal wings. Therefore, interpretation of the digital-modality findings should take these design differences into account rather than relying solely on comparison with the conventional Twin Block.

### 5.4. Comparability of Age and Maturation Between Groups as Potential Confounding Factors

Group comparability with regard to age and maturation stage is particularly relevant in studies of craniofacial growth, as the rate of mandibular growth depends not only on chronological age but also on the stage of skeletal maturation. Although the selection criteria resulted in different age ranges for the treatment and control groups because of the time point at which the age limits were applied (T1 in the treatment group and T2 in the control group), the individual analysis of age showed no statistically significant differences between the two groups. Mean age at T1 was 12.58 years in the treatment group and 12.44 years in the control group (*p* = 0.309), and at T2, it was 14.23 and 14.24 years, respectively (*p* = 0.971), as shown in Figure 5. No statistically significant age differences were observed in the sex-stratified analyses either.

However, the duration of the T1–T2 observation interval was significantly shorter in the treatment group than in the control group (1.65 vs. 1.80 years; *p* = 0.004), a difference that was particularly evident in the male subgroup. For this reason, observation-interval duration, together with age at T1, was included as a covariate in the multivariable sensitivity analyses.

Skeletal maturation represents an additional consideration. Evidence based on cervical vertebral maturation (CVM) indicates that the skeletal response to functional treatment may vary according to maturation stage, particularly during the circumpubertal period. In the present study, a homogeneous indicator of skeletal maturation was not available for all individuals in both groups; therefore, direct comparability using CVM or an equivalent index could not be established. Consequently, although the multivariable analyses adjusted for chronological age, sex, baseline cephalometric value, and observation-interval duration, residual confounding related to differences in skeletal maturation stage cannot be completely excluded. This issue should be considered when interpreting the results and constitutes a limitation of the study.

### 5.5. Clinical Translation: Indications, Patient Compliance, and Stability

From a clinical perspective, comparative literature suggests favourable overall efficiency for removable functional appliances, with Twin-Block-type designs among the most efficient options in terms of correction achieved per unit of treatment time. Indeed, the Twin Block has shown one of the highest efficiency coefficients among functional appliances evaluated in recent systematic reviews. However, these reviews also highlight the scarcity of long-term follow-up data, particularly regarding the stability of changes after the active treatment phase.

In the present study, some of the between-group differences were accompanied by standardised effect sizes of moderate-to-large magnitude. However, the statistical magnitude of an effect does not necessarily imply clinical relevance, and this study did not prespecify thresholds of clinical importance for the variables evaluated. Therefore, the findings should be interpreted by jointly considering the magnitude of the differences, their confidence intervals, and their potential clinical relevance.

Furthermore, the findings correspond to the end of the treatment and observation period and therefore do not establish the long-term stability of the changes or their stability after retention. This is particularly relevant in functional treatment during growth, where some of the observed changes may be related both to treatment and to craniofacial growth itself.

Finally, the clinical characteristics of the different appliance modalities should be considered in practice. Acrylic-splint and clear-aligner variants may differ in aspects such as patient cooperation, soft-tissue tolerance, possibilities for maxillary expansion, and integration into a digital workflow. These characteristics may influence appliance selection and clinical indications. However, because the present study was not designed to compare the two modalities directly, no conclusions regarding differences in efficacy, tolerance, or treatment compliance can be drawn from these findings.

### 5.6. Limitations

The present study has several limitations that should be considered when interpreting its findings. First, its retrospective, non-randomised design, together with the use of a control group derived from historical records in the AAOF Craniofacial Growth Legacy Collection, limits control of potential confounding factors and does not permit causal relationships to be established with the same degree of certainty as a prospective randomised design. In addition, the historical origin of the control group entails potential differences in radiographic acquisition conditions, centres, equipment, and source populations relative to the treatment group.

Second, although no statistically significant age differences were observed between groups at T1 or T2, a homogeneous indicator of skeletal maturation, such as cervical vertebral maturation (CVM) stage, was not available for all individuals in both groups. Because the rate and pattern of craniofacial growth are related to maturation stage, residual confounding associated with potential differences in skeletal maturation cannot be excluded.

In addition, the T1–T2 observation interval was significantly longer in the control group than in the treatment group, particularly in the male subgroup. To reduce the influence of these and other potential between-group differences, multivariable sensitivity analyses were adjusted for the corresponding baseline cephalometric value, age at T1, sex, and observation-interval duration. However, these statistical adjustments do not completely eliminate the limitations inherent in using a historical control group. In particular, the influence of potential population differences or secular trends in craniofacial growth between the periods and populations from which the two groups were drawn cannot be excluded.

Third, sample size was not established by a formal a priori power calculation but by reference to sample sizes in previously published studies. Therefore, although the sample allowed statistically significant differences to be identified in several variables, a lack of significance in other variables should not necessarily be interpreted as evidence of no between-group difference.

Furthermore, the present study analyses only 3 of the 7 areas defined in the complete cephalometric protocol. Conclusions should therefore be limited to the variables and areas analysed in this study and should not be extrapolated to the entire cephalometric protocol. Future studies may extend the analysis to the remaining areas and specifically evaluate potential differences between the different appliance modalities.

Finally, the findings correspond to the end of the treatment and observation period, and no long-term follow-up or post-retention data are available. Consequently, this study cannot determine the long-term stability of the observed changes.

### 5.7. Comparison with Other Studies

Regarding sample size, the present study included 120 individuals, a figure comparable to that of some previously published studies on functional treatment. Bishara et al. [14,15] included 126 individuals. Nevertheless, the adequacy of sample size should not be established solely by comparison with previous studies, particularly because no formal a priori power calculation was performed in the present study, as noted among its limitations.

Regarding dentition, the inclusion of individuals with permanent dentition partially distinguishes our sample from several previous studies in which patients with mixed dentition pre-dominated, such as those by Zhang [16] and Mills [17,18]. Bishara et al. [14,15] included individuals at different stages of dentition, whereas other authors did not clearly specify this aspect, as in the research studies by Chumi Terán [1], Sánchez Albero [2], Planas [4] and Flores-Mir [12]. In the overall sample of the present study, 52.5% of participants were female and 47.5% were male. Analyses were performed both for the overall sample and after stratification by sex; however, formal interaction analyses provided no evidence of differences between males and females in treatment-group-associated changes.

Regarding the records used for measurement, several previous studies, including those by Singh and Clark [8], Zhang [16], Mills [17,18], Oliver [19] and Khan [20], used lateral cephalometric radiographs, as in the present study. More recent studies have incorporated three-dimensional CBCT techniques, as in the cases reported by Duan [21], Khurdal [22], Yildirim [23], Li [24] and Kumar [25]. These methodological differences should be considered when comparing the magnitude of cephalometric changes reported across studies.

Treatment duration also varies considerably in the literature, ranging from approximately 7.4 months in the study by Kumar [25] to 15 months in the studies by Mills [16,17], whereas other authors did not clearly specify treatment duration. This heterogeneity is another factor that complicates direct comparison of the magnitude of changes across studies.

There are also important differences in the cephalometric methods used. Much of the previous literature uses traditional analyses such as Björk–Jarabak, Ricketts, Steiner, or McNamara, whereas the protocol used in the present study is based predominantly on linear measurements and landmarks selected for their reproducibility. The complete method comprises 20 linear and 6 angular measurements. Khan [22] described a protocol with a similar distribution, comprising 18 linear and 6 angular measurements. These differences in reference systems and variables should be considered when making quantitative comparisons across studies.

Regarding sagittal findings, Khan [22], Mills [16,17], and Trenouth [26] described a reduced anterior maxillary displacement, a pattern traditionally termed the “headgear effect”. This pattern is consistent with the smaller A-N change observed in the treatment group in the present study, a difference that remained after multivariable adjustment and correction for multiple comparisons.

Trenouth [26] reported an approximately 2.6° reduction in the ANB angle, whereas Kumar [25] also described a reduction of greater magnitude than that observed in the present study. In our overall sample, the unadjusted TG − CG difference in ANB change was −1.29° (95% CI: −1.73 to −0.85), whereas the adjusted difference was −1.04° (95% CI: −1.46 to −0.62). Although these magnitudes are smaller than those reported in some previous studies, the ANB difference was one of the most consistent findings in the present study and remained statistically significant after multi-variable adjustment and correction for multiple comparisons.

Direct comparison of mandibular growth is more complex. Mills [16,17] and Zhang [15] reported substantial increases using oblique measurements such as Co–Gn, which are not directly equivalent to the variables used in our protocol. In the present study, differences initially observed in some sagittal mandibular measurements did not remain significant after multivariable analysis and correction for multiple comparisons. By contrast, increases in S-Go, A-Me, and N-Me were consistent and remained statistically significant after these adjustments. It is noteworthy that Mills [16,17] described a greater contribution of ramus growth than mandibular body growth, a pattern that may be compatible with the predominance of vertical changes observed in our study. Nevertheless, differences between cephalometric methods preclude direct equivalence between these findings.

## 6. Conclusions

In growing patients with permanent dentition, treatment with the Modified Twin Block (TBM) was associated with differences in sagittal and vertical cephalometric changes compared with the historical control group. The most consistent findings were observed for A-N, S-Go, A-Me, N-Me, and ANB; these differences remained after adjustment for the baseline cephalometric value, age, sex, and observation-interval duration, as well as after multiple-comparison correction using the Benjamini–Hochberg procedure.

The smaller A-N change in the treatment group was compatible with a reduced sagittal maxillary displacement, whereas the greater reduction in the ANB angle indicated a decrease in sagittal maxillomandibular discrepancy. Likewise, the greater increases in S-Go, A-Me, and N-Me demonstrated between-group differences in the vertical dimensions evaluated. By contrast, no significant difference was observed in the change in functional occlusal plane inclination.

These results should be interpreted in light of the retrospective, non-randomised study design, the use of a historical control group, the absence of a homogeneous indicator of skeletal maturation, and the lack of long-term follow-up. Accordingly, the findings support future prospective studies to confirm these results, assess their long-term stability, and analyse the remaining areas of the cephalometric protocol.

## Data Availability

The original contributions presented in this study are included in the article. Further inquiries can be directed to the corresponding author.
